# AKT Pathway Affects Bone Regeneration in Nonunion Treated with Umbilical Cord-Derived Mesenchymal Stem Cells

**DOI:** 10.1007/s12013-014-0378-6

**Published:** 2014-11-21

**Authors:** Zhiguo Qu, Shengnan Guo, Guojun Fang, Zhenghong Cui, Ying Liu

**Affiliations:** 1 Department of Orthopaedic Surgery, Siping Hospital Affiliated to China Medical University, Siping, Jilin China; 2 Tuhua Bioengineering Company Ltd, Siping, Jilin China; 3 Department of Stem Cell Clinical Application Centre, Siping Hospital Affiliated to China Medical University, No. 89, Nanyingbin Road, Tiexi District, Siping, 136000 Jilin China

**Keywords:** AKT, hUC-MSC, BMP-2, OPG, BSP, BGP, Nonunion

## Abstract

We have previously grafted human umbilical cord-derived mesenchymal stem cells (hUC-MSCs) with blood plasma to treat rat tibia nonunion. To further examine the biological characteristics of this process, we applied an established hUC-MSCs-treated rat nonunion model with the addition of an inhibitor of AKT. SD rats (80) were randomly divided into four groups: a fracture group (positive control); a nonunion group (negative control); a hUC-MSCs grafting with blood plasma group; and a hUC-MSCs grafting with blood plasma & AKT blocker group. The animals were sacrificed under deep anesthesia at 4 and 8 weeks post fracture for analysis. The fracture line became less defined at 4 weeks and disappeared at 8 weeks postoperatively in both the hUC-MSCs grafting with blood plasma and grafting with blood plasma & the AKT blocker, which is similar to the fracture group. Histological immunofluorescence studies showed that the numbers of hUC-MSCs in the calluses were significantly higher in the hUC-MSCs grafting with blood plasma than those in group with the AKT blocker. More bone morphogenetic protein 2 and bone sialoprotein expression and less osteoprotegerin and bone gla protein expression were observed in the AKT blocker group compared to the hUC-MSCs grafting with blood plasma. AKT gene expression in the AKT blocker group was decreased 50 % compared to the hUC-MSCs with plasma group and decreased 70 % compared to the fracture group, while the elastic modulus was decreased. In summary, our work demonstrates that AKT may play a role in modulating osteogenesis induced by hUC-MSCs.

## Introduction

Delay or nonunion of fractures occurs in approximately 5–10 % of the 5.6 million patients who sustain a fracture annually [1]. Nonetheless, the poor patient outcomes associated with fracture nonunion point to a critical need for more efficacious strategies for bone regeneration [2]. Current surgical protocols include autologous bone grafts from the iliac crest to achieve bony union. However, harvesting such a graft poses risks for complications and does not necessarily guarantee the fusion of fractures [3]. These clinical scenarios underscore the need to develop novel strategies to enhance bone regeneration in the treatment of musculoskeletal disorders.

Mesenchymal stem cells (MSCs) can be isolated from bone marrow and adipose tissues in adult stages and from placenta, umbilical cord (UC) blood, and connective tissue (Wharton’s Jelly) of human UC [4–8]. MSCs can be induced in vitro and in vivo to differentiate into various mesenchymal tissues such as bone, cartilage, muscle, tendon, adipose tissue, and hematopoiesis-supporting stroma [4]. Osteoblastic differentiation of MSCs is a complex, tightly regulated multi-stage process that is critical for proper bone formation and is influenced by a variety of endogenous and environmental factors and multiple signaling pathways [9–11]. The PI3K/AKT pathway is critical and central in regulating bone cells and controlling skeletal mass. The PI3K/AKT signaling pathway also has been shown to regulate osteoclast survival and differentiation [12, 13]. However, the precise mechanism by which AKT regulates the differentiation of osteoclasts remains unknown.

In a previous study, we grafted human umbilical cord-derived mesenchymal stem cells (hUC-MSCs) with blood plasma to treat rat tibia nonunion. We observed a new multi-center bone formation after stem cell transplantation, which suggested that the biological characteristics of hUC-MSCs-treated nonunion were different from the standard fracture healing process. In the present study, using an inhibitor of AKT and a hUC-MSCs-treated rat nonunion model [14], we demonstrate that AKT plays a role in modulating osteogenesis from MSC differentiation.

## Materials and Methods

### Harvesting of UC

Five human equally sized UC were collected after informed consent was obtained from the mothers in accordance with the ethical committee of the Institute of Siping Central Hospital. Informed consent was obtained from all subjects. All studies and laboratory procedures were carried out in Siping hospital affiliated to China Medical University.

From each sample, sections of 8–10 cm of the UCs, otherwise discarded, were internally washed with phosphate buffered saline (PBS) containing 300 U/ml penicillin and 300 μg/ml streptomycin (Gibco, Grand Island, NY) and immediately immersed in Dulbecco’s modified Eagle’s medium–low glucose (DMEM-LG; Gibco) supplemented with 10 % fetal bovine serum (FBS; Gibco), 300 U/ml penicillin, and 300 μg/ml streptomycin. All samples were processed within 12–15 h after collection.

### Isolation and Culture of Adherent Cells from UC [14]

UCs were filled with 0.1 % collagenase (Sigma-Aldrich, St. Louis) in PBS and incubated at 37 °C for 20 min. Each UC was washed with proliferation medium (α-MEM, 10 % fetal bovine serum; Gibco), and the detached cells were harvested after gentle massage of the UC. Cells were centrifuged at 300×*g* for 10 min, resuspended in proliferation medium, and seeded in 25-cm^2^ flasks at a density of 5 × 10^7^ cells/ml. After 24 h of incubation, non-adherent cells were removed, and culture medium was replaced every 3 days. Adherent cells were cultured until they reached 80–90 % confluence.

### Flow Cytometry

To analyze the cell-surface expression of typical protein markers, adherent cells were incubated with the following anti-human primary antibodies: CD31-phycoerythrin (PE), CD45-fluorescein isothiocyanate (FITC), CD90-R-PE, HLA-DR-R-PE (Becton–Dickinson and Company, Franklin Lakes, NJ). Unconjugated markers were reacted with anti-mouse PE secondary antibody (Guava Technologies, Hayward, CA). A total of 10,000 labeled cells were analyzed using a Guava EasyCyte flow cytometer running Guava ExpressPlus software (Guava Technologies).

### Experimental Nonunion Model

80 SD rats (age at 6–8 weeks) were used in this study. All surgical procedures were performed under anesthesia and sterile conditions. Anesthesia was performed with 4 % Halothane inhalation, followed by Ketamine hydrochloride (80 mg/kg) administered intraperitoneally. The rats were divided into four groups (with equal weight distributions): 1-fracture group (*n* = 20) as a positive control; 2-nonunion group (*n* = 20) as a negative control; 3-hUC-MSCs + plasma group (*n* = 20); 4-hUC-MSCs + plasma and AKT blocker group (*n* = 20). AKT blocker used in this study was perifosine. Each rat received 2 μg of blocker by intraperitoneal injection before surgery and 1day, 3day post-surgery. Rats were sacrificed at 4 weeks and 8 weeks by cervical dislocation under deep anesthesia.

Fractures were performed as described previously [14]. Immediately after the fracture, a 1.25-mm-diameter k-wire was inserted from the trochlear groove into the femoral canal in a retrograde fashion with use of a motor-driven drill. A 5 mm incision in the skin was made around the k-wire, and the wire was then cut at the proximal end of the femur. After irrigation, the wounds were closed with a 5-0 nylon suture. In order to produce the nonunion, the fracture site was minimally exposed laterally and the periosteum was cauterized (Loop tip surgical cautery, Abco Dealer Inc. Nashville, TN) circumferentially for a distance of 2 mm on each side of the fracture.

Animals were regularly monitored radiographically. Mediolateral and anterior–posterior radiographs were taken postoperatively and at 28 and 56 days (4 and 8 weeks) after surgery. Five specimens from each time point were randomly selected for biomechanical testing as described below. The five remaining specimens from each group were processed for histological study. If the fracture produced was not a stable transverse fracture or if the evidence of deep infection developed, then the animal was excluded from the study and replaced with another animal.

The study was approved by the institutional animal care and use committee, following all appropriate guidelines.

### hUC-MSC Transplantation

The rats were placed in a supine decubitus on the operation bed; the left thigh was disinfected with iodophor. Stem cells in 4 ml of blood plasma were injected vertically into the fracture site through the skin in front of the thigh with an epidural needle;for the final 2 ml, the needle was gradually drawn back, and the cells were injected circumferentially around the entire fracture site;once the needle was fully withdrawn, the puncture site was wrapped with sterilized dressing. The rats remained in the supine decubitus on the operation bed for another 30 min before being returned to individual cages. Antibiotics were given to prevent infection.

### Histological Evaluation

At the end of the intervals indicated, 20 rats were euthanized with an excess of carbon dioxide gas and used for histological examination. The right femurs were harvested and fixed in 4 % paraformaldehyde in 0.1 M phosphate buffer for 24 h at 4 °C, diluted in ethanol, decalcified with 10 % formic acid in citrate for 4 days at 4 °C, and embedded in paraffin. Paraffin sections at 4 μm thick were cut and stained with toluidine blue for histological observation. Histology was evaluated to confirm that the standard closed fracture model produced normal stages of fracture healing and that the nonunion model in fact produced nonunion.

### Immunofluorescence

Tibias were embedded in paraffin wax after decalcification in buffered EDTA (14.5 %; pH 7.2) for 2 weeks and were sliced into 3-μm-thick sections following the standard method. The slides were rinsed twice in PBS, followed by a rinse in PBS containing 0.25 % triton X-100 (PBS-TX). The sections were incubated overnight in a dark humid chamber at room temperature with rabbit anti-human ANA (US Biological C7150-13B), rabbit anti-human OPG, rabbit anti-rat BMP-2, rabbit anti-human BGP, or rabbit anti-human BSP (Cell Signaling Technology, Inc. MA, US) diluted 1:200 in PBX-TX containing 1 % bovine serum albumin. After several washes in PBS, the sections were incubated for 1 h in a dark humid chamber at room temperature with goat anti-rabbit IgG conjugated to Alexa488 (Molecular Probes/Invitrogen) or anti-rabbit IgG conjugated to Dylight594 (Molecular Probes/Invitrogen) diluted 1:200 in PBS containing 1 % bovine serum albumin. The sections were rinsed several times in PBS, mounted on cover slips in FluoroSave mounting medium and visualized under a Nikon Eclipse800 fluorescent microscope (Nikon Instruments, NY, USA). Stained cells were counted in each slice by three blinded independent observers to assess the proliferation, localization, and differentiation potential of the hUC-MSCs among the groups. DAPI (Molecular Probes, Grand Island, NY) were used as a nuclear counterstain.

### Biomechanical Test

At the end of the experiment, 5 samples in each group were harvested. A three-point bend test was performed to measure maximum load, maximum/elastic radial degree, and rigidity. Bones were stored and tested in 70 % ethanol. Destructive three-point bend tests were performed on an Instron 5543 materials testing load frame (Instron Limited, High Wycombe, Buckinghamshire, UK) using custom built mounts with rounded supports that minimize cutting and shear loads. Bones were positioned horizontally and centered on the custom supports with the anterior surface upward. A load was applied vertically to the mid-shaft with a constant rate of displacement of 0.03 mm/second until fracture. A span of 12 mm was used. Load–displacement curves were plotted and yield load, maximum load, and fracture load determined. Stiffness, the slope of the linear (elastic) part of the load–displacement curve, was calculated by the “least squares” method. Work energy was calculated from the area under the curve at both maximum load and fracture. Elastic stored energy at maximum load was determined by calculating the area of a right-angled triangle with the vertex at the point of maximum load and hypotenuse with a slope equal to that of the linear phase of the load–displacement curve. Elastic stored energy at fracture was similarly calculated but with the vertex of the triangle at the point of fracture. Energy dissipated at maximum load or fracture was calculated by subtracting the elastic stored energy from the work energy at maximum load or fracture. CVs for each parameter were as follows: yield load (9.8 %), maximum load (8.5 %), fracture load (26.6 %), stiffness (13.6 %), the ratio of energy dissipated at maximum load to elastic stored energy at maximum load (25.1 %), and the ratio of energy dissipated prior to fracture to elastic stored energy at fracture (11.0 %).

### Micro-computed Tomography (μCT)

A SkyScan 1076 micro-computed tomography machine was used to image the proximal tibia and femurs of representative samples from different groups. Samples were scanned at 9-μm resolution, and captured images were rendered using machine software.

### Reverse Transcription-Polymerase Chain Reaction (RT-PCR) [15]

To determine the characteristics of AKT gene expression, total RNA from bone tissue for a distance of 2 mm circumferentially on each side of the fracture was first extracted with Trizol Reagent (Invitrogen, Carlsbad, USA) according to the manufacturer’s instructions. To generate cDNA from total RNA, reverse transcription was performed using SuperScript^TM^ II Reverse Transcriptase (Invitrogen, Carlsbad, USA). Expression of the AKT gene in different groups was analyzed by RT-PCR. Primers used to amplify AKT and β-actin are listed in Table 1. All primers were purchased from Sigma Genosys (Hokkaido, Japan).

**Table 1:** Primers used for the quantification of mRNA levels by semi-quantitative reverse transcriptase-polymerase chain reaction (RT-PCR)

Gene	(5′–3′) Forward primer	(5′–3′) Reverse primer	Accession number	Base pairs	Annealing temperature (°C)	Cycle (s)
AKT/PKB	GAGGAGCGGGAAGAGTG	GAGACAGGTGGAAGAAGAGC	Y 15748	672	54	30
β-Actin	GCCAACCGTGAAAAGATG	CCAGGATAGAGCCACCAAT	NM 31144	681	57	30

### Statistical Methods

Data analysis for the RT-PCR, to compare the nonunion tissue and fracture callus tissue RNA profiles, was performed by a paired Student’s *t* test with equal variance. Statistical analyses were carried out on Stata^®^ software (StataCorp LP, College Station, TX). All the experiments were carried out with triplicate samples and repeated at least three times. One-way analysis of variance (ANOVA) was used to compare the mean values according to bone architecture and biomechanical strength in the presence or absence of perifosine.

## Results

### Isolation and Culture of Adherent Cells from UC

All UC samples generated primary adherent cultures with cells displaying an MSC-like phenotype, which is consistent with our previous report [14]. After a 4 days of culturing, these cells grew in colonies and reached confluence after 10–14 days. Most of the cells were spindle-shaped, resembling fibroblasts. After the second passage, adherent cells constituted homogeneous cell layers with an MSC-like phenotype (14). The number of MSCs from UC decreased slightly after freezing and thawing (14). The remaining viable cells were successfully expanded on consecutive days (data not shown).

### Immunophenotypes

All adherent cells derived from UC did not express hematopoietic lineage markers (CD45) and endothelial markers (CD31), HLA-DR (HLA-class II) as assessed by flow cytometry. In addition, the majority of cells expressed high levels of the adhesion marker CD90.

### Histological Analysis

**Fig. 1:**
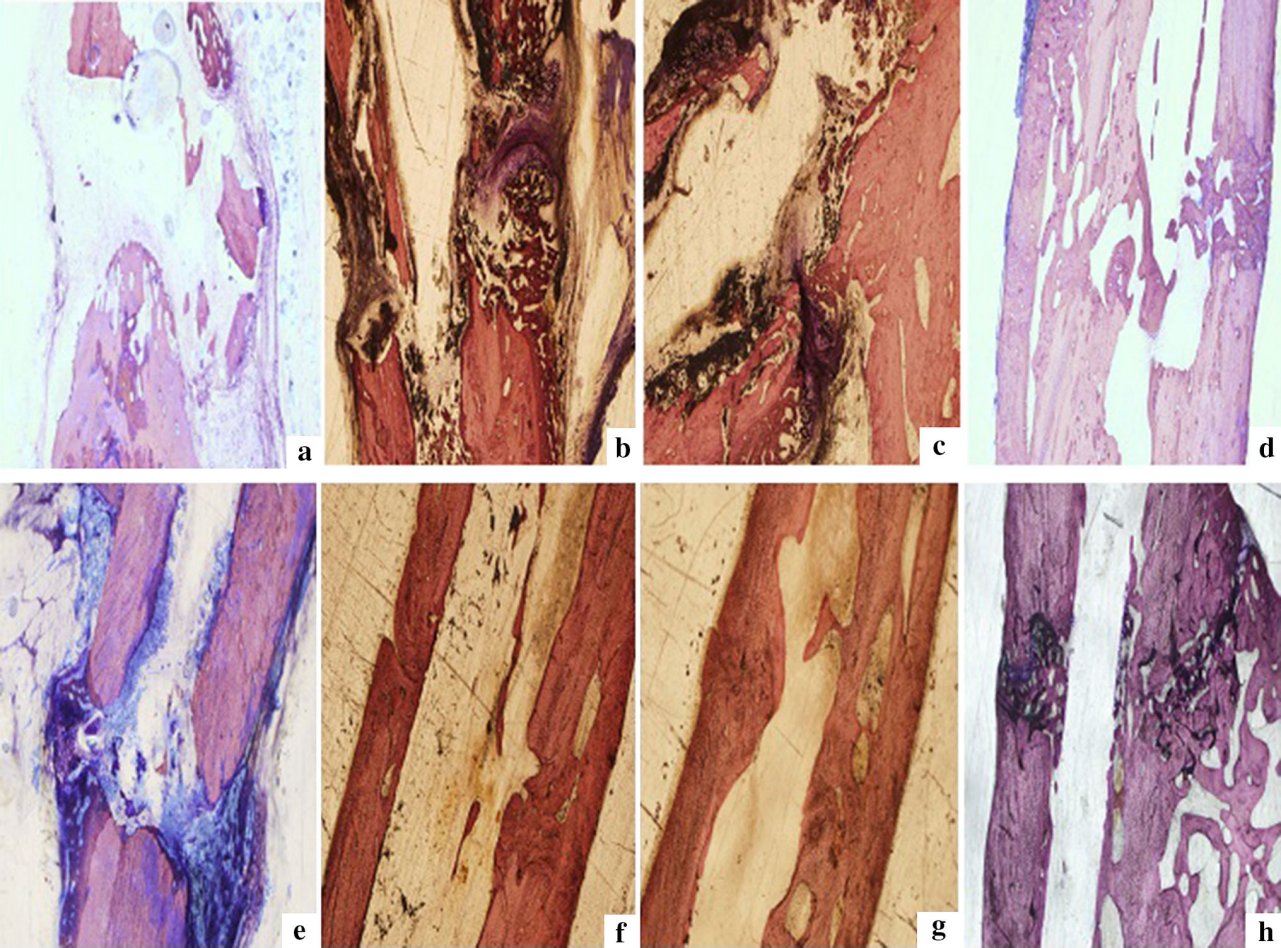
Histological Analysis. **a** 4 Weeks after fracture, the callus in the gap is wide. **b** Intramembranous bone ossification in the periosteum and cartilage fracture. These formed a thick callus of chondrocytes, newly formed trabecular bone, and the two calluses on each side of the fracture are almost uniform. **c** Cartilage and endochondral ossification in hUC-MSCs transplantation + plasma group are similar to the fracture group. **d** The gap between the callus in the hUC-MSCs with plasma + AKT blocker group is less than the gap in the nonunion group, while the callus that formed is thin. **e** At 8 weeks after fracture, there is still a big gap at the end of the woven bone surface in the bone nonunion group and cortical bone resorption has stopped. **f** In the fracture group, the callus connecting the cartilage area has almost disappeared. The fracture is covered, the newly formed trabecular bone has healed, and the woven bone reengineering thickness has gradually decreased. The interface of the primitive cortical bone fracture is not obvious. **g** The fracture coverage by the newly formed trabecular bone in hUC-MSCs transplantation + plasma group is similar to the fracture group, and is healing, but the bone marrow cavity is thin. **h** In the hUC-MSCs transplantation with plasma + AKT blocker group, the gap of the fracture is covered by newly formed trabecular bone, but it has not healed

At 4 weeks after induction of a fracture, the gap between the calluses was wider in the nonunion group (Fig. 1a). The fracture group of rats displayed intramembranous ossification in the periosteal tissue and endochondral ossification at the fracture site (Fig. 1b). A thick callus was formed, which consisted of chondrocytes and newly formed trabecular bone. The two calluses in each side of the fracture were almost joined. The gap between endochondrocytes and endochondral ossification in the group who received stem cell grafts with blood plasma was similar to those observed in the fracture group (Fig. 1c), but there was no bone formation on the site of periosteal cauterization. In addition, in the group who received stem cells grafts with plasma and AKT blocker, the gap between the calluses was smaller than that of the hUC-MSCs and plasma group, and the callus formed was thin. The united bone in fracture group with joined bone had remodeled with a progressive decrease in the nests of the woven bone (Fig. 1d). In contrast, a large gap persisted between the surfaces of woven bone in the rats with nonunion (Fig. 1e). 8 weeks after fracture induction, the callus in the fracture group had joined and chondrogenic areas almost disappeared (Fig. 1f). The fractured bone was covered with newly formed trabecular bone and achieved bony union. In the stem cell grafting with blood plasma group, similar to the fracture group, the fractured bone was covered with newly formed trabecular bone and achieved bony union but the bone marrow cavity was thinner (Fig. 1g). However, in the nonunion model at 8 weeks, the fibrous tissue surrounded the fracture site and resorption of the end of the cortical bone had started (Fig. 1e). This was consistent with the histological presentation of atrophic nonunions. In the stem cell grafting with plasma and AKT blocker group, the fractured bone was covered with newly formed trabecular bone but it had not yet achieved bony union (Fig. 1h). Notably, at 8 weeks, the fracture group with united bone had remodeled with a progressive decrease in the thickness of the woven bone (Fig. 1h). The fracture gap at the interface of the original cortical bone was not detectable.

### Immunofluorescence Findings

**Fig. 2:**
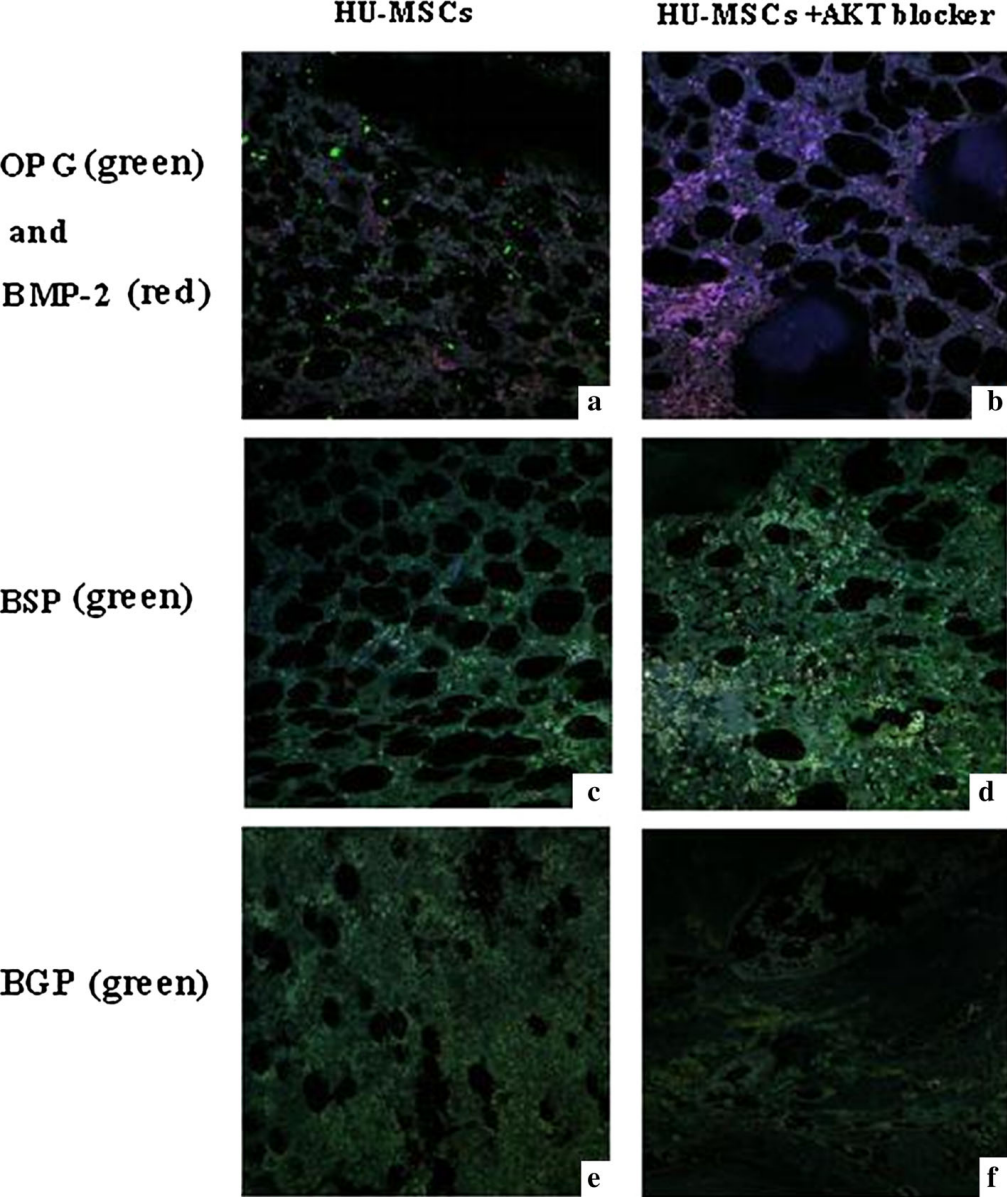
Immunohistological Findings. Biological characteristics of hUC-MSCs with/without AKT blocker at 8 weeks after fracture. **a** OPG and BMP-2 expression in the hUC-MSCs transplantation with plasma group; **b** OPG and BMP-2 expression in the hUC-MSCs transplantation with plasma and AKT blocker group; **c** BSP expression in hUC-MSCs transplantation with plasma group; **d** BSP expression in the hUC-MSCs transplantation with plasma and AKT blocker group; **e** BGP expression in the hUC-MSCs transplantation with plasma group; **f** BGP expression in the hUC-MSCs transplantation with plasma and AKT blocker group

To further study the biological characteristics of hUC-MSCs, we examined anti-human OPG and anti-rat BMP-2 staining in calluses of osteotomized rat tibiae in the stem cell transplantation groups at 8 weeks after fracture (Fig. 2) (Note: hUC-MSCs were identified by green fluorescence in Fig. 2a). In the calluses of osteotomized rat tibiae, large numbers of labeled cells were observed. Moreover, the numbers of labeled cells in these calluses were significantly higher (data were not shown.) than those of the hUC-MSCs transplantation with plasma and AKT blocker group (Fig. 2b). No anti-human OPG-labeled hUC-MSCs expressed bone morphogenetic protein BMP-2, which is visible as red fluorescence in the calluses of osteotomized rat tibiae (Fig. 2a, b). We also examined anti-human BSP and BGP expression in both the hUC-MSCs transplantation with plasma with or without AKT blocker groups. We observed more BSP and less BGP expression in the hUC-MSC transplantation with plasma and AKT blocker group compared with that in hUC-MSCs transplantation with plasma group.

### Micro-computed Tomography (μCT)

**Fig. 3:**
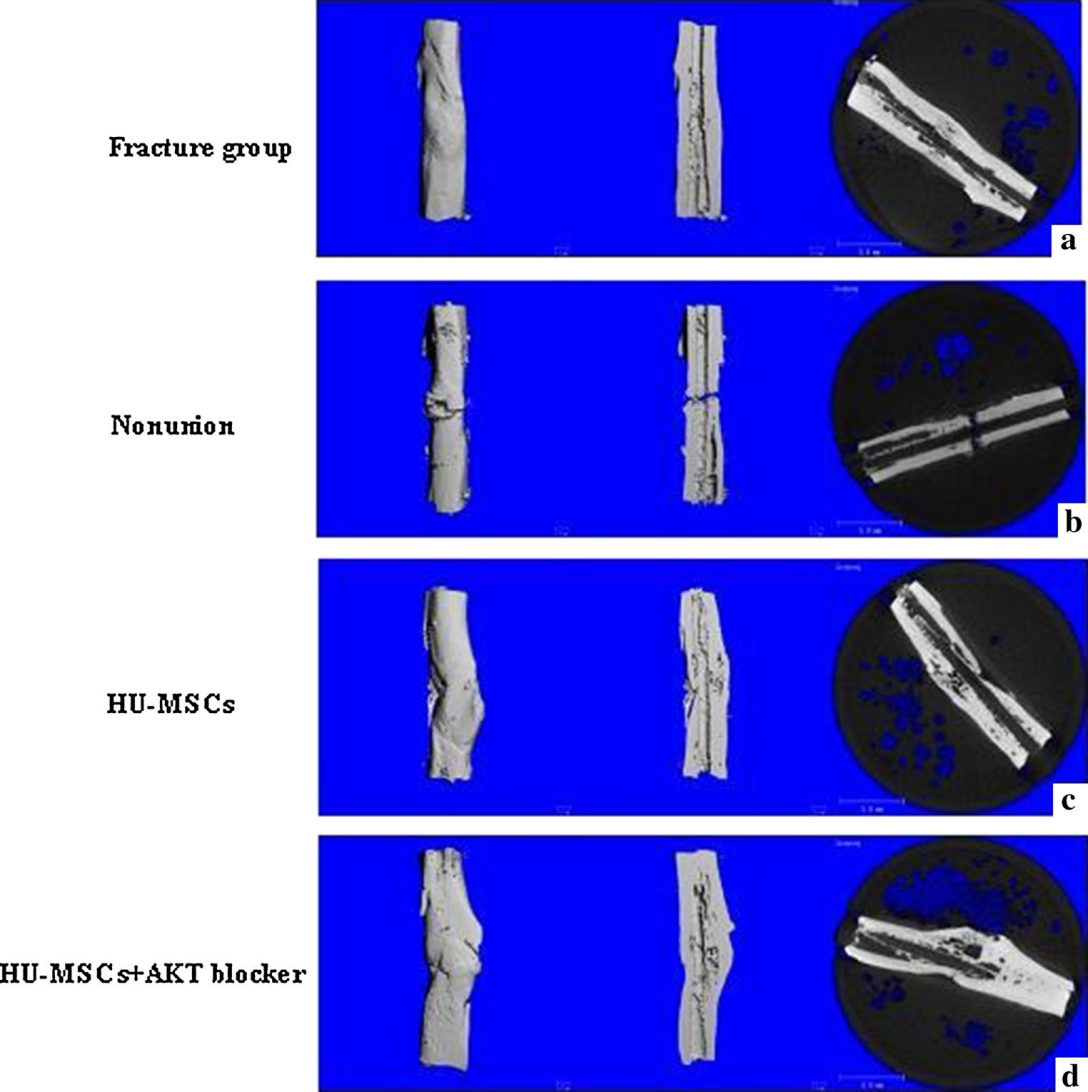
Representative tibial uCT scans. **a** Fracture group rat, **b** Nonunion group rat, **c** Nonunion rat treated with hUC-MSCs + plasma and **d** Nonunion rat treated with hUC-MSCs + plasma + AKT blocker

Figure 3 shows representative μCT scans of tibiae from different groups. Compared to the fracture group, rat tibiae in the nonunion group have clear morphological abnormalities that include a widened epiphysis, disruption of the growth plate, and large fissures that are likely occupied by unmineralized osteoid tissue in the living animal (compare Fig. 3b with a). In contrast, the hUC-MSCs + plasma treated rat tibiae show a clear and marked improvement and the treated bone compares favorably with the rat tibia in the nonunion group (compare Fig. 3c with b). The improvements likely reflect an increase in mineralization and a reduction in osteoid tissue in the hUC-MSCs + plasma treated animals. Figure 3 proposes a model to explain how the AKT inhibitor improves the bone formation in rats of the hUC-MSCs + plasma & AKT blocker group (compare Fig. 3d with b) by imaging analysis compared with the hUC-MSCs + plasma group.

### Expression of AKT Genes

**Fig. 4:**
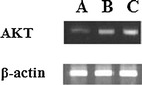
Expression of AKT genes. *A* Expression of AKT genes in the hUC-MSCs + plasma + AKT blocker group; *B* Expression of AKT genes in the hUC-MSCs + plasma group; *C* Expression of AKT genes in the fracture group as a positive control

To further characterize the AKT expression in the fracture group, the bone tissues all around the fracture and for a distance of 2 mm on each side of it were isolated from, the fracture group, the hUC-MSCs + plasma and hUC-MSCs + plasma & AKT blocker groups at 8 weeks post-surgery. The AKT expression in the hUC-MSCs + plasma & AKT blocker group was decreased 50 % compared to the hUC-MSCs + plasma group and decreased 70 % compared to the fracture group (Fig. 4).

### Biomechanical Study on the Correlation Between hUC-MSC Transplantation for Treating Rat Bone Nonunion and AKT

To further characterize the AKT effect in the fracture group, 5 rats in each of the hUC-MSCs + plasma group and hUC-MSCs + plasma & AKT blocker groups at 8 weeks after transplantation were used in a rat tibial bending test. The elastic modulus decreased in the hUC-MSCs + plasma & AKT blocker group compared to the hUC-MSCs + plasma group (Table 2).

**Table 2:** hUC-MSC transplantation for treating rat bone nonunion; biomechanical study on the correlation with AKT

	Fracture group	hUC-MSCs + blood plasma	hUC-MSCs + blood plasma + AKT blocker	*p* value
Bending stress value at the maximum bending load (MPa)	525.14 ± 65.36	84.69 ± 32.17	49.99 ± 16.21*^△^	<0.05
The bending strain in the maximum bending load (%)	11.70 ± 3.22	20.73 ± 6.51	31.66 ± 5.47*^△^	<0.05
Geometry	Round	Round	Round	
Pivot span (mm)	10.00	10.00	10.00	
Diameter (mm)	2.00	3.00	4.00	
Speed (mm/min)	2.00	2.00	2.00	
The bending displacement in the maximum bending load (mm)	0.97 ± 0.26	1.15 ± 0.31	1.32 ± 0.53*^△^	<0.05
The maximum bending load (N)	164.98 ± 23.55	89.79 ± 19.76	125.65 ± 21.42*^△^	<0.05
Modulus of elasticity (Automatic Young’s) (MPa)	15903.14 ± 366.79	2253.54 ± 121.66	589.24 ± 116.34*^△^	<0.01

*p: The values of hUC-MSCs + blood plasma + AKT blocker compared to these of fracture group; ^△^ *p*: The values of hUC-MSCs + blood plasma + AKT blocker compared tothese of fracture group

## Discussion

Previous studies in our lab with hUC-MSCs have shown that co-administration of blood plasma enhances osteogenesis of these stem cells by increasing bone markers and calcium mineral deposition [14]. However, it is still an ongoing challenge to mimic natural bone and engineer functional, weight-bearing bone tissue with hUC-MSCs treatment. In the present study, we further defined the biomechanical properties of osteogenesis from hUC-MSCs during this process.

BMP-2 is reported to increase bone formation both in vitro and in vivo [16–18]. Addition of BMP-2 vastly increases osteocalcin [19] and a short-term expression of BMP-2 is necessary and sufficient to irreversibly induce bone formation [20]. In our current study, with the co-administration of blood plasma plus hUC-MSCs with/without AKT inhibitor groups, we found that the expression of BMP-2 increased in the transplanted stem cells and the surrounding tissue. The results suggest that the transplanted hUC-MSCs may secrete soluble factors that could stimulate local cells and hUC-MSCs themselves to express BMP-2, which further stimulates the transformation of transplanted stem cells into osteocytes and promotes the healing of the fracture. BMP-2 has been reported to regulate stem cell proliferation and differentiation [21, 22]. Our data also suggest that the proliferation of the transplanted hUC-MSCs may benefit from BMP-2 in a positive feedback mechanism.

Differentiation of hUC-MSCs into osteocytes requires the involvement of intrinsic signaling pathways. Among the intracellular signaling pathways, the phosphoinositide 3-kinase and AKT (protein kinase B) signaling pathway (PI3K/AKT) plays a central role in the control of cell survival, growth, and proliferation throughout the body. In addition, PI3K/AKT is also reported as a central nexus in the extensive network of extracellular signaling pathways that control osteoblasts [23]. However, the biological effects of AKT on the osteo-differentiation of hUC-MSCs remain an underexplored area of investigation. In the current study, we observed a larger volume of moved bone in the AKT inhibitor group with decreased biomechanical strength. These results show an imbalance in the bone remodeling process, and bone fragility leading to bone fractures upon applying biomechanical stress. Our data may contribute insight to the mechanism of osteoporosis, which is characterized by low bone density and deterioration of bone micro-architecture [24]. Tsuji et al. reported that the earliest steps of fracture healing seem to be blocked in bones lacking BMP2 [25]. In our study, we did not observe obvious differences in the expression of BMP-2 in both stem cell transplantation groups. This, together with the finding of reduced OPG expression in the AKT inhibitor group, confirms a cell autonomous regulation of OPG by AKT in hUC-MSCs transdifferentiation and raises the possibility that AKT directly controls the osteo-differentiation of hUC-MSCs. In the current study, we identified that the expression of AKT is decreased in hUC-MSCs-treated bone tissues. However, the mechanism for the deceased AKT expression is still unclear. One recent report suggested that the AKT blocker perifosine, in addition to AKT activity inhibition, may also inhibit AKT expression [26]. Another possibility for the deceased AKT expression might be induced by indirect effect of perifosine on the functions hUC-MSCs.

In summary, our work demonstrates that co-administration of blood plasma plus hUC-MSCs accelerates the healing of fracture nonunion and that AKT may play a role in modulating osteogenesis from MSC differentiation. These results are consistent with activation of tissue repair in both transplanted hUC-MSCs and the host bone. In addition, these findings reinforce our previous suggestion on the importance of banking the whole UC unit for research or future therapeutic use.
